# Peptide-HLA-based immunotherapeutics platforms for direct modulation of antigen-specific T cells

**DOI:** 10.1038/s41598-021-98716-z

**Published:** 2021-09-28

**Authors:** Ronald D. Seidel, Zohra Merazga, Dharma Raj Thapa, Jonathan Soriano, Emily Spaulding, Ahmet S. Vakkasoglu, Paige Ruthardt, Wynona Bautista, Steven N. Quayle, Peter A. Kiener, Simon Low, John F. Ross, Saso Cemerski, Anish Suri, Steven C. Almo, Rodolfo J. Chaparro

**Affiliations:** 1 Cue Biopharma, Cambridge, MA USA; 2 BioKien LLC, Harpswell, ME USA; 3 Albert Einstein College of Medicine, Bronx, NY USA

**Keywords:** Biotechnology, Immunology

## Abstract

Targeted pharmacologic activation of antigen-specific (AgS) T cells may bypass limitations inherent in current T cell-based cancer therapies. We describe two immunotherapeutics platforms for selective delivery of costimulatory ligands and peptide-HLA (pHLA) to AgS T cells. We engineered and deployed on these platforms an affinity-attenuated variant of interleukin-2, which selectively expands oligoclonal and polyfunctional AgS T cells in vitro and synergizes with CD80 signals for superior proliferation versus peptide stimulation.

## Introduction

There remains a significant need for immunotherapeutics to drive clinically effective anti-tumor T cell responses. Adoptive T cell therapies (ACT) have demonstrated impressive clinical results for several cancers using patient-derived T cells activated ex vivo with potent T cell receptor (TCR), costimulatory and cytokine stimulation^1^. However, cellular therapies reach few patients due to complex manufacturing and reinfusion requirements, patient conditioning regimens and safety considerations. In contrast, systemic costimulation represents a scalable pharmacologic approach to cancer immunotherapy which has the intent of activating and expanding tumor-specific T cells directly within the patient. However, attempts to induce anti-tumor T cell responses using systemic agonism with agents such as anti-CD137 antibodies and high dose interleukin-2 (IL-2) are associated with significant risk of toxicity^2,3^. IL-2 is of particular interest as a potent cytokine capable of inducing the proliferation and differentiation of CD8 effector T cells (Teff), as well as other T, B, and NK lineages with anti-tumor potential. In the contexts of metastatic renal cell carcinoma and malignant melanoma, high-dose IL-2 can induce curative remissions in a minority of patients, associated with elevated levels of AgS Teff, but is dose limited by severe and potentially life-threatening toxicities such as vascular leak syndrome and cytokine release syndrome^4^. The anti-tumor effects of IL-2 are also indirectly limited by regulatory T cells (Treg), which expand efficiently to IL-2 in vivo due to high level expression of the high affinity IL-2 receptor. Elevated Treg counts, which can limit Teff responses, are associated with poor prognosis in cancer patients^5^. In addition, cancer immunotherapeutic combinations targeting distinct costimulatory pathways hold the potential of greatly amplifying T cell responses^6^. However, as illustrated by the combined inhibition of CTLA-4 and PD-1 pathways in the treatment of metastatic melanoma, toxicities may also compound, limiting their utility^7^. Broader therapeutic use of IL-2 and other costimulatory axes in cancer will likely require focusing their effects on those T cells which stand to deliver the greatest therapeutic effect, in particular cancer AgS T cells.

Cancer vaccines represent another scalable pharmacologic approach to cancer immunotherapy. However, vaccine efficacy depends on antigen-presenting cell (APC) function, including trafficking, antigen-processing, costimulation versus co-inhibition, and susceptibility to tumor immunosuppression^8^. For example, dendritic cells are APC which play critical roles in the priming and maintenance of anti-tumor CD8 T cell responses via delivery of peptide-HLA and potent costimulatory ligands such as CD80, CD86, and CD137L^8^. However, dendritic cells are also replete with inhibitory ligands such as TIM-3, PD-L1, PD-L2, HVEM, B7-H3, B7-H4, IL-T3, and IL-4, which blunt T cell responses and are subject to local and distal tumor influence^9^. Therefore, safe, scalable and APC-independent immunotherapeutics are needed that will enable clinically effective levels of anti-tumor T cell activation and associated tumor cell killing. We have identified a potential solution to this challenge based on the natural signals governing T cell function: peptide-HLA and costimulatory ligands, embodied in the Immuno-STAT (Selective Targeting and Alteration of T cells) and Neo-STAT immunotherapeutics platforms. Immuno-STAT and Neo-STAT utilize compact, Fc-based architectures amenable to clinical manufacturing, and are designed to focus optimized signals for potent costimulatory axes such as IL-2 directly to AgS T cells in vivo, thereby enhancing anti-tumor T cell responses while avoiding indiscriminate immune activation.

## Results

**Figure 1:**
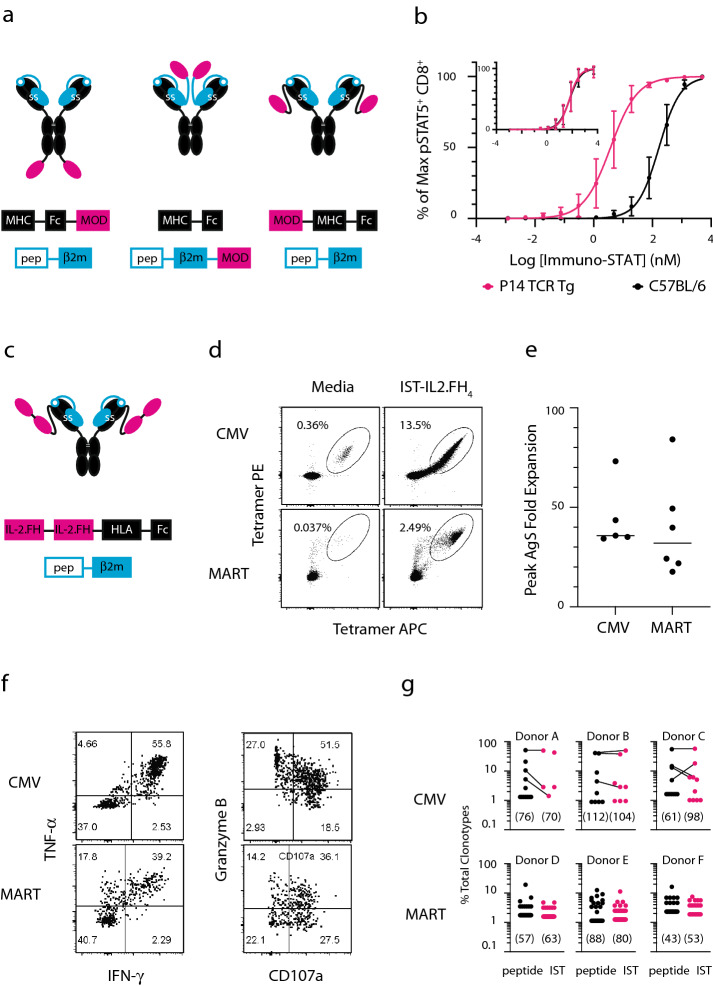
Immuno-STAT design, optimization and activity. (**a**) Immuno-STAT frameworks comprising peptide (pep) epitope, β2m, MHC heavy chain, Fc, and co-modulatory domain (MOD) in different relative positions, covalently linked by engineered (ss) and native ( =) disulfide bonds. (**b**) In vitro pSTAT5 activity of P14 transgenic versus C57BL/6 CD8 splenocytes challenged with construct LCMV-IST-IL2.FH_4_. Inset: Fc fusion bearing four IL2.FH but without pMHC. %pSTAT5^+^ responses within each independent dose response titration were normalized as: normalized response = (sample − minimum)/(maximum − minimum). Data represent mean $\pm$ SD of duplicate samples from three independent experiments. (**c**) Humanized Immuno-STAT framework comprising two copies of pHLA, four copies of IL2.FH and human IgG1 Fc. (**d**) Representative dual-tetramer plots of human PBMC stimulated in vitro for ten days with media, CMV-IST-IL2.FH_4_, or MART-IST-IL2.FH_4_. (**e**) Peak fold expansion of AgS T cells from human PBMC incubated with CMV-IST-IL2.FH_4_ (n = 5 donors) and IST-MART-IL2.FH_4_ (n = 6 donors), from 1 to 4 independent expansions per donor. (**f**) Representative IFN-γ, TNF-α, CD107a, and granzyme B staining following cognate peptide challenge of Immuno-STAT-expanded CMV- or MART-specific T cells. (**g**) Paired TCRαβ sequence frequencies from individual CMV- or MART-specific CD8 T cells expanded with cognate Immuno-STAT or peptide. Lines connect Immuno-STAT- and peptide-expanded clones having TCRαβ sequence identity. Three donors per specificity. Total clones surveyed in parentheses.

The Immuno-STAT framework comprises a covalent fusion of peptide epitope, MHC class I allele, co-modulator, and Fc, which imparts avidity and symmetrical multivalency, sufficient for cognate T cell activation (Fig. 1a)^10^. Potential co-modulators, costimulatory and co-inhibitory ligands, may be fused to the N- or C-terminus of the MHC-Fc heavy chain or the C-terminus of β2m, allowing organizational, compositional and stoichiometric flexibility. Our initial exploration of the Immuno-STAT platform utilized IL-2 as the co-modulator to activate and expand AgS cytotoxic effector T cells (Teff).

To identify an optimized IL-2-based Immuno-STAT framework, we evaluated a panel of constructs for relative potency, AgS selectivity, and manufacturability. Constructs comprised LCMV gp_33-41_/H-2D^b^, recognized by the murine TCR P14, fused at its N-terminus to variants of human IL-2 and C-terminally to an effector-attenuated murine IgG2a Fc. IL-2-attenuating mutations were included to limit IL-2Rα-dependent toxicity and Treg engagement as well as to reduce IL-2 affinity in favor of pMHC selectivity (see Supplementary Fig. 1)^11,12^. IL-2 stoichiometries were limited to two or four based on manufacturability which showed a significant drop in protein titer beyond four copies of IL-2 (see Supplementary Fig. 2). Human IL-2 exhibits potent activity on both human and mouse cells including phosphorylation of STAT5, an IL-2 receptor (IL-2R) proximal signaling molecule and phosphorylated STAT5 (pSTAT5) serves as an index of IL-2R engagement which correlates well with downstream consequences of IL-2R agonism such as proliferation and phenotypic marker expression^12^. We compared pSTAT5 induction for purified CD8 splenocytes from AgS P14 TCR transgenic mice to non-AgS C57BL/6 mice and ranked constructs based on logEC50_P14_ (potency index), P14 minus C57BL/6 signal at EC50_P14_ (selectivity index), and protein expression titer (see Supplementary Fig. 3 and Supplementary Table 1). Construct LCMV-IST-IL2.FH_4_ ranked highest followed by LCMV-IST-IL2.F_4_, bearing four copies of IL-2^F42^^A, H16A^ or IL-2^F42A^, respectively. Dose responses for top candidates LCMV-IST-IL2.FH_4_ and LCMV-IST-IL2.F_4_ from this initial screen were reevaluated over a broader concentration range and with greater resolution and compared with reference constructs bearing two or four copies of wildtype IL-2, LCMV-IST-IL2_2_ or LCMV-IST-IL2_4_, respectively (Fig. 1b and see Supplementary Fig. 4). AgS potency, as indicated by the logarithm of the pSTAT5 EC50 for responding P14 splenocytes (logEC50_P14_), was not significantly different between LCMV-IST-IL2.FH_4_, LCMV-IST-IL2.F_4_, LCMV-IST-IL2_2_ and LCMV-IST-IL2_4_ (see Supplementary Table 2 and Supplementary Fig. 5). Likewise, non-AgS potency (logEC50_B6_) was also similar for these constructs (see Supplementary Table 2 and Supplementary Fig. 5). AgS selectivity was measured first by the difference in normalized pSTAT5 signal at EC50_P14_ for AgS (P14) splenocytes (i.e. 50%) relative to non-AgS (C57BL/6) splenocytes as well as by the difference in the logEC50 for AgS versus non-AgS splenocytes (see Supplementary Fig. 6). Both measures of AgS selectivity were not significantly different across constructs LCMV-IST-IL2.FH_4_, LCMV-IST-IL2.F_4_, LCMV-IST-IL2_2_ and LCMV-IST-IL2_4_. Thus, significant F42A- and H16A-mediated decreases in IL-2R signaling potency or selectivity were not observed relative to reference constructs bearing two or four copies of wildtype IL-2 (LCMV-IST-IL2_2_ or LCMV-IST-IL2_4_), presumably masked in part by the increased IL-2 stoichiometry.

We next generated and tested humanized Immuno-STATs bearing IL2.FH_4_, effector-attenuated human IgG1, HLA-A*0201 and model epitopes CMV pp65_495-503_ or MART1_26-35_ (Fig. 1c and see Supplementary Fig. 7). For multiple donors, we determined the frequency of dual-tetramer-positive AgS CD8 T cells following a 10 day incubation of human PBMC with specific IST-IL2.FH_4_ (Fig. 1d and see Supplemental Fig. 8). CMV-IST-IL2.FH_4_, but not CMV-IST (no IL-2 fusion) was able to expand CMV-specific CD8 T cells from PBMC, indicating that proliferation was dependent on the presence of IL2.FH_4_ (see Supplementary Fig. 9). Similarly, CMV-IST in the presence, but not absence, of recombinant human IL-2 (wildtype)-expanded CMV-specific CD8 T cells in vitro, further supporting the requirement for IL-2R agonism for Immuno-STAT activity (data not shown). Peak expansions (> 30 fold) were similar for PBMC CD8 T cells responsive to CMV-IST-IL2.FH_4_ or MART-IST-IL2.FH_4_ (Fig. 1e). In addition, peak expansions for PBMC CD8 T cells responsive to CMV-IST-IL2.FH_4_ or MART-IST-IL2.FH_4_ were achieved at similar Immuno-STAT concentrations (see Supplementary Fig. 10). However, higher CMV- versus MART-specific frequencies were present in expanded and media cultures (see Supplementary Fig. 10). The cytokines IFN-γ, TNF-α, granzyme B and the membrane protein CD107a are phenotypic markers whose combinatorial expression by lymphocytes is associated with cytotoxic function in multiple contexts, including clinically effective anti-tumor responses^13^. A significant fraction of Immuno-STAT- and peptide-expanded AgS CD8 T cells expressed IFN-γ, TNF-α, granzyme B and CD107a in response to cognate peptide challenge (Fig. 1f and see Supplementary Fig. 11). Responses to irrelevant peptide were negligible for IFN-γ, TNF-α and CD107a (see Supplementary Fig. 12). Responses to irrelevant peptide for granzyme B were above zero but generally below responses to cognate peptide for all concentrations measured (see Supplementary Fig. 12). Importantly, Immuno-STAT-expanded AgS CD8 T cells expressed multiple of these phenotypic markers simultaneously, similar to peptide-expanded AgS CD8 T cells and consistent with differentiation of a potent Teff population (Fig. 1f and see Supplementary Fig. 12). Dose responses were also similar for Immuno-STAT- and peptide-expanded AgS CD8 T cells expressing IFN-γ, TNF-α, granzyme B and CD107a (see Supplementary Fig. 12). Consistent with robust Teff repertoires, tetramer-sorted single cells from peptide or Immuno-STAT expansions showed similar TCRαβ oligoclonal frequency distributions and sequence-identity was observed between a subset of IST- and peptide-expanded CMV-, but not MART-reactive clones, which were predominantly low clonality (Fig. 1g).

**Figure 2:**
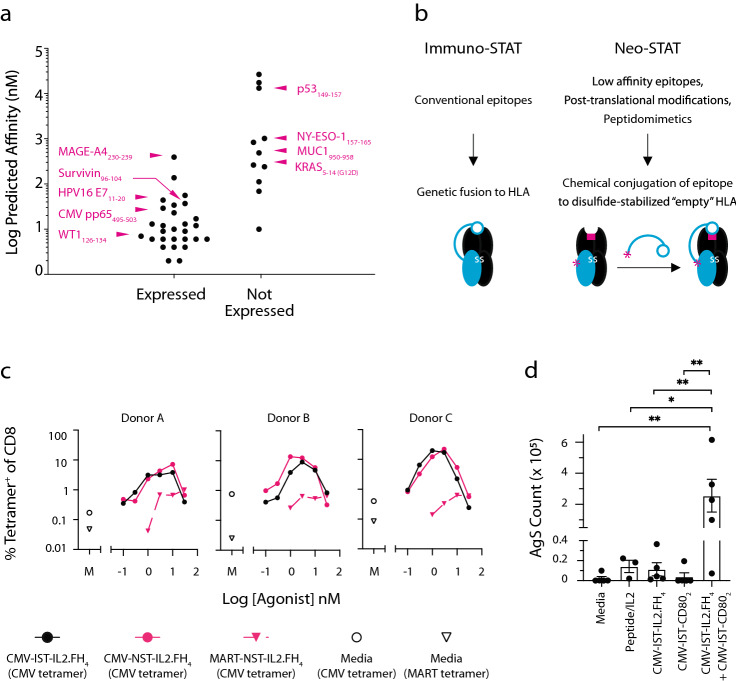
Immuno-STAT and Neo-STAT epitope and co-modulatory flexibility. (**a**) NetMHC-predicted peptide binding affinity to HLA-A*0201 versus expressibility on the Immuno-STAT framework, highlighting select epitopes. Data from two independent expression trials with minimum expressibility criteria of 10 mg/L and 50% species of interest by SDS-PAGE. (**b**) Differential application of Immuno-STAT and Neo-STAT frameworks by epitope type. (**c**) Dual-tetramer frequency of CMV-specific human PBMC T cells following ten day culture with CMV Neo-STAT (CMV-NST-IL2.FH_4_), MART-Neo-STAT (MART-NST-IL2.FH_4_), CMV-IST-IL2.FH_4_, or media (M). Data are single measurements at each concentration for three donors. (**d**) CMV-specific human PBMC T cell counts following ten day culture of CMV-IST-IL2.FH_4_-responsive donors with CMV-IST-IL2.FH_4_ and/or CMV-IST-CD80_2_ versus peptide and media controls. Data represent single measurements per condition from 3 to 5 donors. Statistical significance *(*P* $\le$ 0.05) or **(*P* $\le$ 0.01) assessed by one-tailed paired ratio T test.

The modular Immuno-STAT framework is compatible with diverse co-modulators, peptides and HLA alleles. For example, IL-2, CD80, CD86 and CD137L represent potent costimulatory ligands for CD8 T cells, variants of which can be integrated into the Immuno-STAT framework (see Supplementary Fig. 13). Furthermore, beyond HLA-A*0201-associated peptides, AFP_403-411_/HLA-A*1101 and HBV Protein P_109-118_/HLA-A*2402, well characterized cancer and infectious disease antigens, respectively, express well on the Immuno-STAT framework without the comodulator (i.e. pHLA only, see Supplementary Fig. 13). In our hands expression of the Immuno-STAT pHLA is highly predictive of expression of Immuno-STAT pHLA-comodulator combinations such as Immuno-STAT-IL2.FH_4_. However, epitopes with weak binding to HLA are generally more challenging to express, including clinically significant cancer epitopes such as KRAS G12D (Fig. 2a and see Supplementary Table 3). We therefore developed Neo-STAT, which uses site-specific chemical conjugation of peptides to a stably engineered “empty” HLA, to enable presentation of low-affinity peptides and moieties not accessible via genetic fusion (Fig. 2b). CMV pp65_495-503_ and MART1_26-35_, model epitopes previously explored on the Immuno-STAT framework, were successfully conjugated to empty HLA-A*0201 on the Neo-STAT framework bearing IL2.FH_4_ (i.e. NST-IL2.FH_4_, see Supplementary Fig. 14). CMV-NST-IL2.FH_4_ selectively expanded CMV-specific T cells with similar potency to CMV-IST-IL2.FH_4_ (Fig. 2c). Next, as an initial exploration of co-modulator flexibility and the potential of combinatorial co-modulation, we assessed the ability of CMV-IST bearing the CD80 ectodomain (CMV-IST-CD80_2_) to induce antigen-specific proliferation in vitro, both as a single agent and when combined with CMV-IST-IL2.FH_4_. Whereas CMV-IST-CD80_2_ alone did not induce AgS proliferation above background levels, CMV-IST-CD80_2_ demonstrates synergy with CMV-IST-IL2.FH_4_ in expanding CMV-specific T cells in vitro and at levels superior to peptide stimulation (Fig. 2d).

## Discussion

We have described two modular immunotherapeutics platforms, Immuno-STAT and Neo-STAT, for the co-presentation of defined peptide-HLA (pHLA) and co-modulatory ligands to AgS T cells, and engineered an affinity-attenuated variant of IL-2, IL2.FH, for use thereon. IL2.FH-bearing constructs, including an HPV-specific clinical candidate, CUE-101 (NCT03978689), are associated with 110-fold and 3.1-fold decreases in binding to IL2Rα and IL2Rβ, respectively, with minimal Treg and non-AgS T cell responses in vitro and in vivo^14^. While greater AgS selectivity was anticipated for LCMV-IST-IL2.FH_4_ over LCMV-IST-IL2_2_ due to attenuation of IL-2:IL-2R versus pHLA:TCR binding, this trend was not statistically significant. Potentially more selective variants (e.g. F42A D20K) affecting critical contacts with IL-2Rβ were associated with low peak activities, suggesting trade-offs between selectivity and Rmax^15,16^. Integration of Immuno-STAT signals through the TCR and IL-2R and/or more IL-2R distal events such as cytokine release may reflect greater selectivity than pSTAT5 induction. Proliferation showed greater selectivity versus pSTAT5 induction for AgS CD8 T cells in response to IST-IL2.FH_4_, extending similar results obtained with CUE-101. In addition, through modestly attenuated IL-2Rβ binding, H16A may enable strong, but submaximal IL-2 signals, thereby limiting burnout/terminal differentiation while driving proliferation, effector function and memory T cell generation^16,17^.

AgS cytotoxic CD8 T cell responses have previously been achieved via heterologous expression of membrane pMHC complexes, for example, by DNA vaccination^18^. Alternatively, nanoparticle display of pMHC can stimulate or inhibit AgS T cell responses depending on the presence and nature of costimulatory and co-inhibitory ligands^19,20^. In contrast, soluble pMHC complexes, including streptavidin-based tetramers, largely suppress T cell responses^21,22^. To our knowledge, Immuno-STAT and Neo-STAT molecules are the first embodiments of soluble pMHC-comodulator signaling complexes directed to AgS T cells in cis.

Immuno-STAT and Neo-STAT access diverse HLA alleles and epitopes in compact architectures which may benefit clinical manufacturability and biodistribution^23^. HLA-A*0201, HLA-A*1101 and HLA-A*2402, surveyed here in the context of Immuno-STAT, are collectively expressed by over half the global population and Neo-STAT extends potential pHLAs to include post-translational modifications and low affinity peptides^24^. In preclinical murine studies, CUE-101 and mCUE-101 elicit AgS T cell responses from both naïve and antigen-experienced precursors, with responses to mCUE-101 detectable in blood, spleen, and tumors, suggesting the potential to both invigorate T cell responses within tumors and initiate responses outside of immunosuppressive tumor microenvironments, which are relatively resistant to exhaustion^14,25^. Compelling applications of these platforms include enhancing T cell responses against essential antigens in cancer or infectious disease, especially where relevant AgS T cells are limiting or dysfunctional, and more efficiently activating or maintaining ACT cells ex/in vivo. In addition, conjugating clinical grade peptides and Neo-STAT precursors enables a rapid and parallelizable manufacturing paradigm for immunotherapeutics capable of addressing neoantigens, multi-epitope combinations to address tumor heterogeneity and mitigate tumor escape and rapid responses to emerging pandemics.

As shown here, distinct Immuno-STAT co-modulators can synergize for superior AgS T cell activation in vitro. Moreover, unlike current vaccines, Immuno-STATs and Neo-STATs may bypass APC in vivo to drive therapeutic T cell responses. The same core architectures bearing appropriate comodulators may also be used to engage other costimulatory pathways, such as CD137 and CD70^26,27^. Likewise, Immuno-STATs and Neo-STATs may be used to engage or block inhibitory pathways such as PD-1 and CTLA-4 for AgS activation or downregulation, respectively, while limiting toxicity^7^. Whereas the present work has focused on classical HLA class I restricted T cells, alternative versions of these scaffolds may, in principle, be used to engage any AgS T cell, including T cells specific for non-classical HLA class I ligands as well as HLA class II-restricted ligands.

## Materials and methods

### Design, manufacturing, purification, and characterization of Immuno-STAT and Neo-STAT proteins

Immuno-STAT proteins bearing H-2D^b^ were genetically fused to an effector-attenuated murine IgG2a^28^. Immuno-STAT and Neo-STAT proteins bearing human HLAs were fused to an effector-attenuated human IgG1 and linked to CMV pp65_495-503_ or MART1_26-35_, immunodominant epitopes in the context of human cytomegalovirus infection or malignant melanoma^29–31^. Domains within the Immuno-STAT and Neo-STAT frameworks are linked via G_4_S linkers. Cysteine substitutions R12C of β2m and A236C of HLA-A*0201 result in a stabilizing disulfide bond between these polypeptides. Neo-STAT also contains a disulfide bond comprising cysteine substitutions at Y84C and A139C of HLA-A*0201, which stabilizes the “empty” (i.e. epitope-less) Neo-STAT precursor prior to conjugation with the peptide epitope of interest^32^. An engineered cysteine, E44C, within β2m serves as the attachment site for maleimide conjugated peptides.

Immuno-STAT and Neo-STAT proteins were expressed by transient transfection in Expi-CHO cells (ThermoFisher). Proteins were purified from the conditioned media using a two-step method of ProteinA capture with MabSelect SuRe (GE) followed by size exclusion chromatography. For SDS-PAGE analysis, proteins were boiled in SDS sample buffer with or without reducing agent for 5 min before loading 2 µg per gel lane.

Empty Neo-STAT precursor proteins were linked to maleimide-conjugaged peptides using standard maleimide chemistry. Briefly, empty Neo-STAT precursors were exchanged into and partially reduced with a TCEP-based reducing buffer before two rounds of conjugation with a 20 fold molar excess of peptide-maleimide in the absence of TCEP. Conjugated Neo-STAT proteins were washed at low pH to remove excess unconjugated peptides in solution before purification by size exclusion and mass confirmation by electrospray ionization time of flight mass spectrometry (ESI-TOF MS).

### Animals studies

Spleens were collected from C57BL/6J and P14 T cell receptor (specific for LCMV gp_33-41_/H-2D^b^) transgenic mice (Jackson Labs) at least six weeks of age following euthanasia by CO_2_ inhalation and confirmation of euthanasia by cervical dislocation. All studies requiring animal tissues were approved by the Institutional Animal Care and Use Committee for SmartLabs (Cambridge, MA) and were performed in compliance with federal guidelines and in accordance with the ARRIVE guidelines 2.0.

### pSTAT5 assay and phosphoflow analysis

Spleens were harvested from C57BL/6J and P14 T cell receptor (specific for LCMV gp_33-41_/H-2D^b^) transgenic mice (Jackson Labs). Splenocytes from 10 to 15 mice (depending on the number of conditions tested) per strain were pooled and CD8 T cells were isolated using Dynabeads Untouched Mouse CD8 kit (ThermoFisher). CD8 splenocytes were resuspended in RPMI culture media (ATCC) supplemented with 10% fetal bovine serum (Hyclone). 1 × 10^5^ C57BL/6J CD8 splenocytes or P14 TCR Tg CD8 splenocytes were seeded in 96-well U-bottom plates and stimulated with Immuno-STAT at a given concentration per well for 20 min at 37 °C (initial Immuno-STAT-IL2 framework variant screen) or 5 min at 37 °C (follow up studies) in a final volume of 100 µl. For the initial Immuno-STAT framework variant screen, mouse splenic CD8 T cells were incubated with Immuno-STAT proteins at 0.01, 0.1, 1, 10, 100, 250, 500 and 1000 nM. For confirmatory studies of LCMV-IST-IL2.FH_4_, LCMV-IST-IL2.F_4_, LCMV-IST-IL2_4_, LCMV-IST-IL2_2_, IL2.FH_4_-Fc and rhIL-2 (Peprotech), mouse splenic CD8 T cells were incubated with test articles at 0.00119, 0.00477, 0.0191, 0.0763, 0.305, 1.22, 4.88, 19.5, 78.1, 313, 1250, and 5000 nM. Cells were immediately fixed with IC fixation buffer (ThermoFisher), permeabilized with 100% methanol, and stained with a 1/10 dilution of anti-pY694 STAT5 antibody (BD Biosciences) for 30 min at room temperature, followed by flow cytometry on an iQue Screener (Intellicyt) for initial IST variant screen or an Attune NxT cytometer (Invitrogen) for follow up studies. The percent of positively stained cells was determined using FlowJo software (TreeStar). Non-linear curve fits and EC50 determinations from pSTAT5 dose response data were performed using Prism analysis software (Graphpad).

### Human T cell expansion, tetramer staining and flow cytometry analysis

Human healthy donor peripheral blood mononuclear cells (PBMC) were obtained as frozen stocks (Astarte Biologics) or isolated from leukopaks (HemaCare); washed and resuspended in ImmunoCult-XF Cell Expansion Media (Stemcell Technologies). 1 × 10^7^ PBMC were seeded in a 6 well plate with specific Immuno-STAT or Neo-STAT at a given concentration or media control in a total volume of 4 ml. Cells were incubated with 0, 0.1, 1, 3, 10, 30, or 100 nM CMV-IST-IL2.FH_4_ or MART-IST-IL2.FH_4_, with each concentration evaluated in 1–4 independent expansion trials. 0.3 nM Immuno-STAT was also evaluated for CMV-IST-IL2.FH_4_. For Neo-STAT expansion studies, PBMC were incubated with CMV-IST-FH_4_ or CMV-NST-FH_4_ at 0, 0.1, 0.3, 1, 3, 10 and 30 nM and PBMC were incubated with control MART-NST-IL2.FH_4_ at 0, 1, 3, 10 and 30 nM. For experiments examining the combined activity of CMV-IST-IL2.FH_4_ and CMV-IST-CD80_2_, cells were incubated with CMV-IST-IL2.FH_4_ and CMV-IST-CD80_2_ at 1 and 100 nM, respectively, or with media alone or with 5 µg/ml CMV pp65_495-503_ (NLVPMVATV) peptide plus 50 IU/ml IL-2. Immuno-STAT or peptide expansion cultures were maintained in a 37 °C CO_2_ incubator with replacement of half the culture media at day 5 and day 7. Cells were harvested on day 10, washed, resuspended in FACS buffer on ice and stained for viability using Fixable Viability Stain 780 (BD Biosciences) before staining with relevant tetramers (MBL International, MA) on ice for 30 min. Tetramer-stained cells were then washed and stained on ice with antibodies against CD3 (clone SK7, BioLegend), CD14 (clone M5E2, BioLegend), CD19 (clone HIB19, BioLegend), CD56 (clone HCD56, BioLegend), CD4 (clone SK3, BioLegend) and CD8 (clone SK1, BD Biosciences) for 30 min. Tetramer flow cytometric data was acquired using the Attune NxT cytometer (ThermoFisher) and analyzed using FlowJo software (Tree Star). Peak fold expansion per donor per trial was calculated as the maximum tetramer-positive frequency observed for PBMC expanded with specific IST-IL2.FH_4_ over tetramer-positive frequency for media-incubated PBMC. Peak fold expansion data was derived from between one and three expansion trials per donor, with mean peak fold expansion values used for donors with multiple expansion trials.

### Intracellular cytokine and phenotypic staining of in vitro-expanded human CD8 T cells

A total of 2 to 4 × 10^6^ human PBMCs expanded with specific Immuno-STAT or peptide were pretreated with brefeldin A (BFA) and monensin (ThermoFisher), plated in a 24-well plate, and stimulated at a 1:1 ratio with T2 cells (ATCC) that had been loaded with CMV pp65_495–503_ (NLVPMVATV) or Mart1_26–35_ (ELAGIGILTV) or HIV-1 p17 Gag_77–85_ (SLYNTVATL; SL9) peptide for 2 h and washed twice. Cells were stimulated for 5 h, washed, stained with Fixed Viability Stain 780 (BD Biosciences), antibodies against CD3 (clone SK7, BioLegend), CD8 (clone SK1, BD Biosciences) and CD107a (clone H4A3, BD Biosciences), and fixed using IC fixation buffer (ThermoFisher). Cells were next washed in permeabilization buffer (eBioscience), stained with antibodies against TNF-α (clone MAb11, BD Biosciences), IFN-γ (clone 4S.B3, BioLegend), and granzyme B (clone GB11, BD Biosciences) for 30 min at room temperature, washed, and analyzed. For representative FACS plots and pairwise marker quantitation, PBMC were stimulated as described above using T2 cells loaded with 100 nM peptide. For peptide titration studies, PBMC were stimulated as described above using T2 cells loaded with 1 × 10^–13^, 1 × 10^–12^, 1 × 10^–11^, 1 × 10^–10^, 1 × 10^–9^, 1 × 10^–8^, 1 × 10^–7^, 1 × 10^–6^, or 1 × 10^–5^ g/ml peptide.

### TCR sequencing

PBMCs from healthy donors were expanded in vitro with CMV pp65_495-503_ or Mart1_26-35_ peptide plus IL-2, or with 100 nM specific Immuno-STAT in 10-day cultures. Expanded cells were harvested, tetramer stained, and CMV- or MART1-specific CD8^+^ T cells were single cell sorted (Sony SH800) and the α and β TCR chains were coamplified (iRepertoire). TCR library sequencing used an Illumina MiSeq v2 Nano Kit. Data for each individual well were demultiplexed, mapped, and analyzed using the iRmap VDJ pipeline and the iPair Analyzer.

## Supplementary Information

Supplementary Information.
