# Supplementary material for: Peptide-HLA-based immunotherapeutics platforms for direct modulation of antigen-specific T cells
Source: Sci Rep. 2021 Sep 28;11:19220. doi: 10.1038/s41598-021-98716-z (PMC8479091; doi:10.1038/s41598-021-98716-z)
Supplement: Supplementary file 1 — Supplementary Information. [file 41598_2021_98716_MOESM1_ESM.pdf]

Peptide-HLA-based immunotherapeutics platforms for direct modulation of antigen-specific T cells

Ronald D. Seidel[1*], Zohra Merazga[1], Dharma Raj Thapa[1], Jonathan Soriano[1], Emily Spaulding[1], Ahmet Vakkasoglu[1], Paige Ruthardt[1], Wynona Bautista[1], Steven N. Quayle[1], Peter A. Kiener, Simon Low[1], John F. Ross[1], Saso Cemerski[1], Anish Suri[1], Steven C. Almo[2], Rodolfo J. Chaparro[1]

Author affiliations:

[1]Cue Biopharma, Cambridge, Massachusetts

[2]Albert Einstein College of Medicine, Bronx, New York

*Correspondence should be addressed to R.D.S. seidel.ron@gmail.com

Supplementary Figures

Supplementary Figure 1

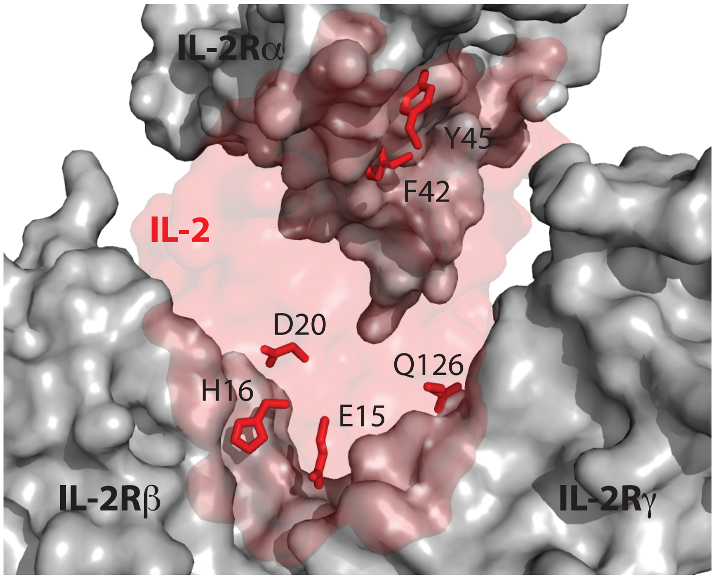

Supplementary Figure 1. IL-2 mutations affecting IL-2Rαβγ subunit interactions explored in Immuno-STAT variant screen. IL-2 shown in space-filled transparency with side-chains corresponding to mutations of interest (solid red wire frame) docked with IL-2Rα, IL-2Rβ, and IL-2Rγ subunits (gray space-filling model). Crystallographic data for model derived from PDB 2ERJ.

Supplementary Figure 2

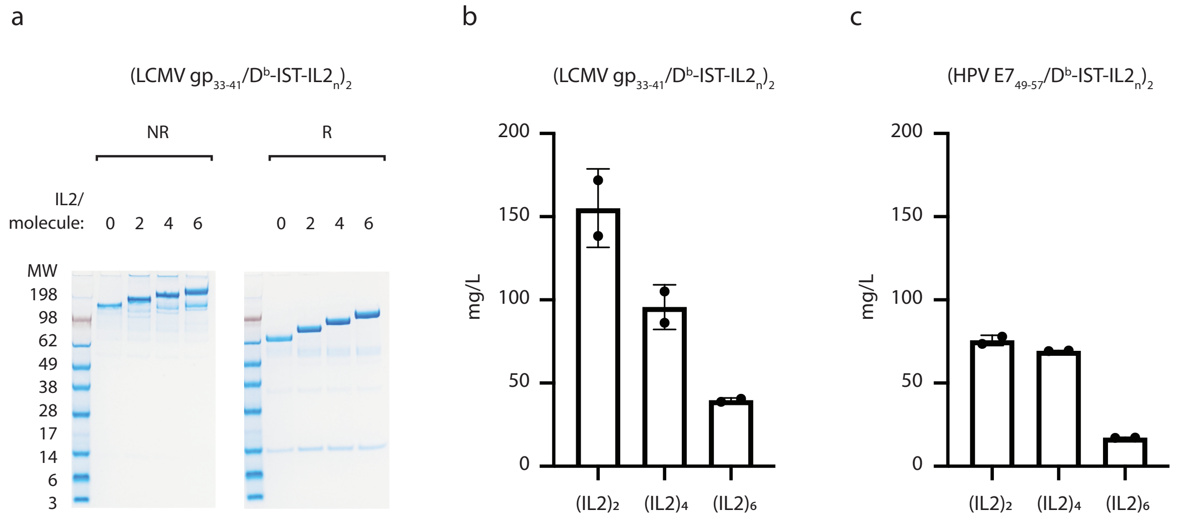

Supplementary Figure 2. Decrease in Immuno-STAT protein expression with increasing stoichiometry of IL-2. (a) Reducing (R) and non-reducing (NR) SDS-PAGE of (gp33/D^b-IST-$IL2_n$)$_2$ bearing 0, 2, 4 or 6 copies of wild-type IL-2 per molecule. (b) Protein expression titers for (gp33/D^b-IST-$IL2_n$)$_2$ bearing 2, 4 or 6 copies of wild-type IL-2 per molecule. (c) Protein expression titers for IST-(HPV $E7_{49-57}$/D^b-IST-$IL2_n$)$_2$ bearing 2, 4 or 6 copies of wild-type IL-2 per molecule. Data in (b) and (c) represent duplicate expression samples.

Supplementary Figure 3

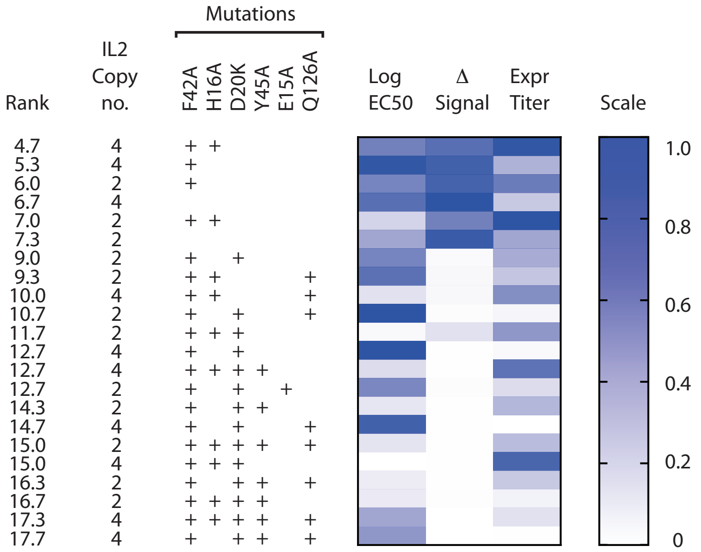

Supplementary Figure 3. Initial screen of stoichiometric and mutational variants of IL-2 genetically fused to the N-terminus of the MHC heavy chain on the Immuno-STAT framework, showing composite rank and heat map. Composite rank based on the averaged ranks of the log EC50 for pSTAT5 induction of P14 TCR Tg CD8 splenocytes, the difference in pSTAT5 signal for P14 TCR Tg vs C57BL/6 CD8 splenocytes as determined at the P14 EC50 (Δ Signal), and the expression titer. Log EC50, Δ Signal, and expression values normalized to 1 for heat map graphical representation. Data represent mean values of duplicate cell samples in pSTAT5 activity assays and duplicate or triplicate protein expression titers.

Supplementary Figure 4

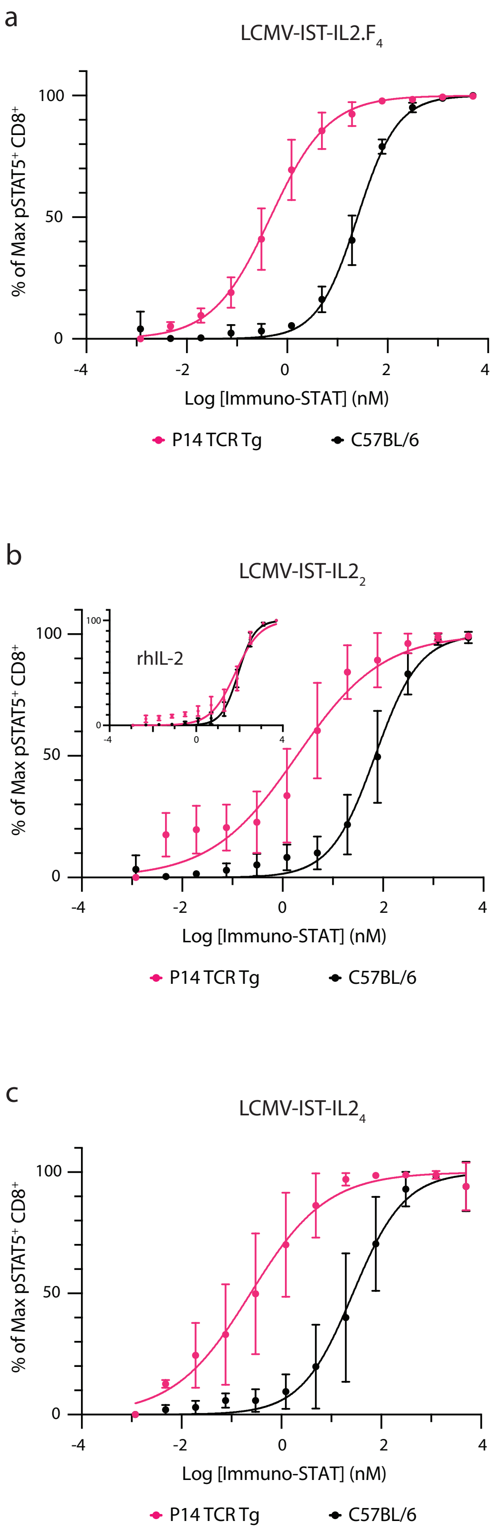

Supplementary Figure 4. Confirmation of in vitro pSTAT5 activity for LCMV-IST-IL2.F_4, LCMV-IST-$IL2_2$ and LCMV-IST-$IL2_4$. In vitro pSTAT5 activity of P14 TCR transgenic versus C57BL/6 CD8 splenocytes challenged with Immuno-STAT comprising (a) two LCMV $gp_{33\text{-}41}$/H-$2D^b$ and four variant IL-2 (F42A) per molecule (LCMV-IST-IL2.F_4) or (b) Immuno-STAT comprising two LCMV $gp_{33\text{-}41}$/H-$2D^b$ and two wild type IL-2 per molecule (LCMV-IST-$IL2_2$) with recombinant human IL-2 inset or (c) Immuno-STAT comprising two LCMV $gp_{33\text{-}41}$/H-$2D^b$ and four wild type IL-2 per molecule (LCMV-IST-$IL2_4$). %$pSTAT5^+$ responses within each independent dose response titration were normalized as: normalized response = (sample – minimum)/(maximum – minimum). Data represent mean $\pm$ SD of duplicate samples from three independent experiments.

Supplementary Figure 5

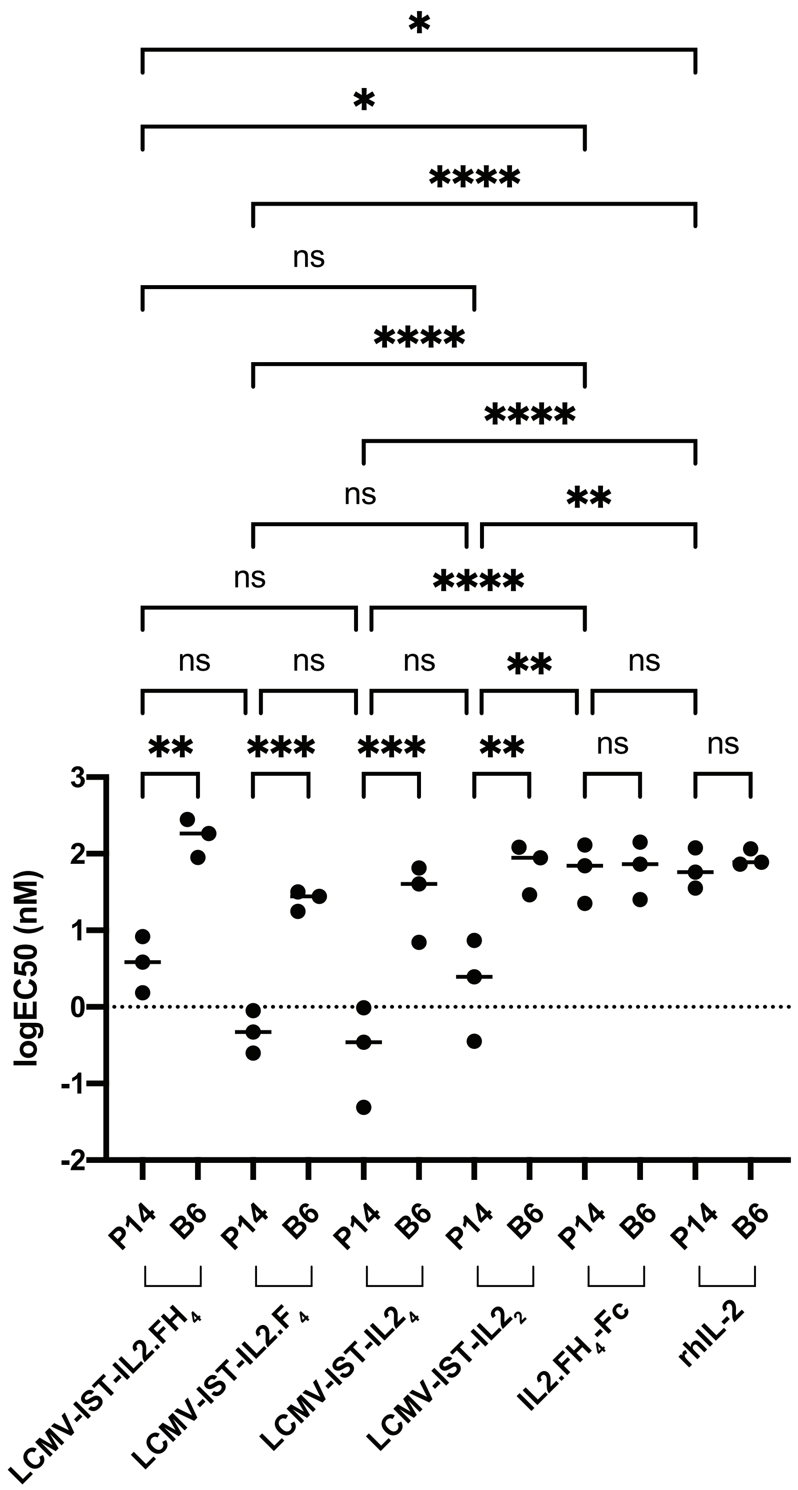

Supplementary Figure 5. AgS potency. Comparison of logEC50 from pSTAT5 dose responses in P14 TCR Tg and C57BL/6 splenocytes for top-ranked Immuno-STAT LCMV-IST-IL2.FH_4 and second-ranked Immuno-STAT LCMV-IST-IL2.F_4 versus wildtype IL-2 reference constructs LCMV-IST-$IL2_4$, LCMV-IST-$IL2_2$ and untargeted IL-2 controls: recombinant human IL-2 and an IL2.FH_4-Fc fusion protein lacking pMHC. LCMV-IST-IL2.FH_4 comprises two LCMV gp_{33-41}/H-$2D^b$ and four IL-2(F42A, H16A) per per molecule. LCMV-IST-IL2.F_4 comprises two LCMV gp_{33-41}/H-$2D^b$ and four IL-2(F42A) per per molecule. LCMV-IST-$IL2_4$ and LCMV-IST-$IL2_2$ comprise two LCMV gp_{33-41}/H-$2D^b$ bearing four and two wild-type IL-2 per per molecule, respectively. %$pSTAT5^+$ responses within each independent dose response titration were normalized as: normalized response = (sample – minimum)/(maximum – minimum). The EC50s were derived in Graphpad PRISM from the best fit equation for the normalized dose response data. A one-way ANOVA using Tukey's multiple comparisons test was used to assess statistically significant differences between test articles; $P \leq 0.05$ (*), $P \leq 0.01$ (**), $P \leq 0.001$ (***), $P \leq 0.0001$ (****), $P > 0.05$ non-significant (ns).

Supplementary Figure 6

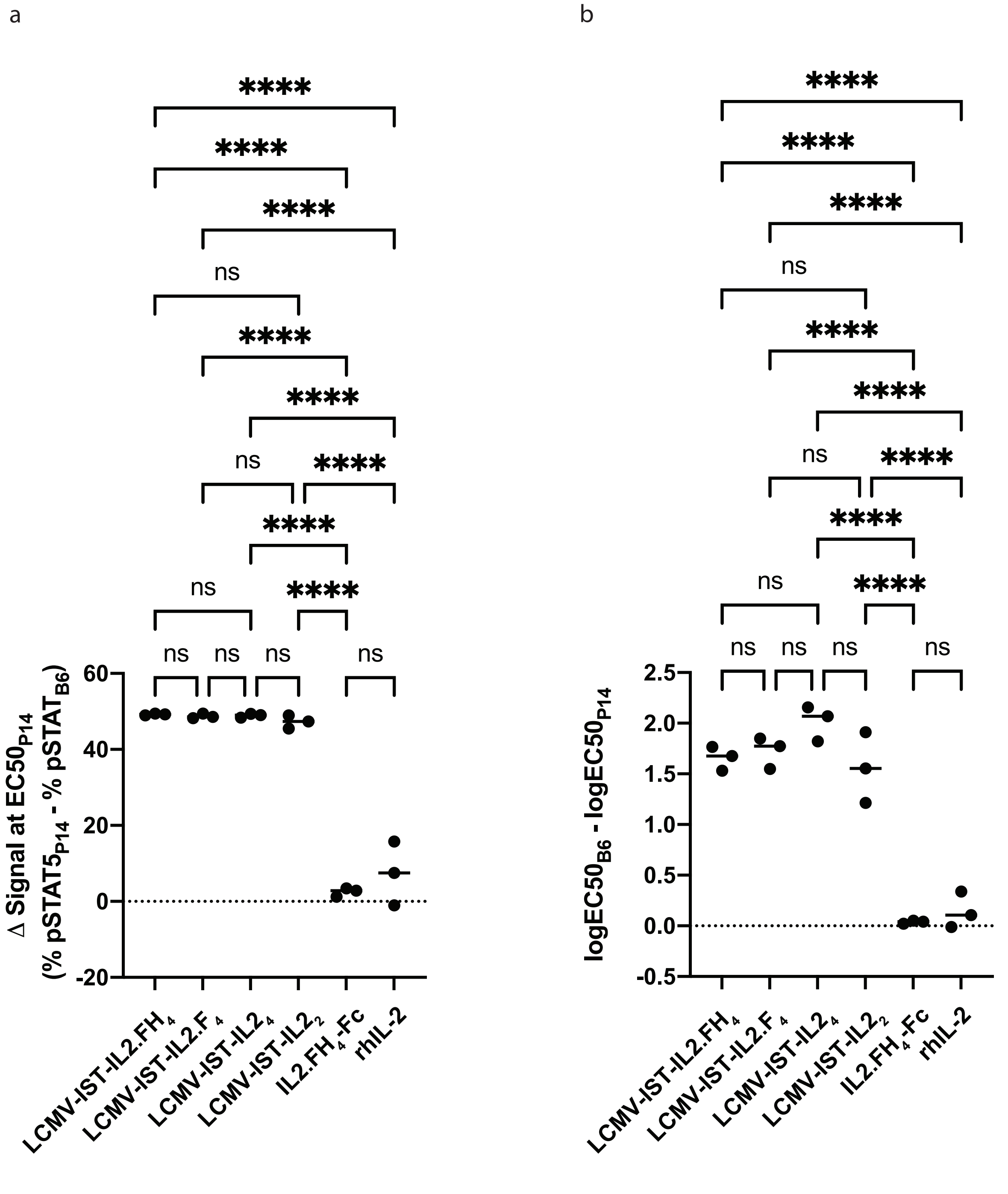

Supplementary Figure 6. AgS selectivity. Top-ranked Immuno-STAT-IL2 framework variant LCMV-IST-IL2.FH_4 and second-ranked LCMV-IST-IL2.F_4 were compared to wildtype IL-2

reference constructs LCMV-IST-$IL2_4$, LCMV-IST-$IL2_2$, untargeted IL-2 controls (recombinant human IL-2 and an IL2.FH_4-Fc fusion protein lacking pMHC) with respect to (a) the difference in pSTAT5 signal between P14 TCR Tg and C57BL/6 CD8 splenocytes at the EC50 determined for P14 TCR Tg CD8 splenocytes. Data are from duplicate samples from three independent experiments. %$pSTAT5^+$ responses within each independent dose response titration were normalized as: normalized response = (sample – minimum)/(maximum – minimum). The EC50s were derived in Graphpad PRISM from the best fit equation for the normalized dose response data. The pSTAT5 signal from C57BL/6 CD8 splenocytes was calculated by evaluating the best fit equation for the C57BL/6 CD8 splenocyte pSTAT5 dose response at the P14 EC50. A one-way ANOVA using Tukey's multiple comparisons test was used to assess statistically significant differences between test articles; $P \leq 0.0001$ (****), $P > 0.05$ non-significant (ns). (b) Groups as in (a) but comparing the difference in pSTAT5 logEC50 between P14 TCR Tg ($logEC50_{P14}$) and C57BL/6 ($logEC50_{B6}$) CD8 splenocytes. Data and statistical comparisons as in (a).

Supplementary Figure 7

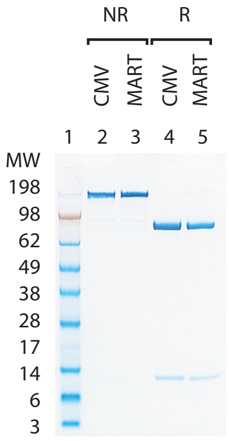

Supplementary Figure 7. Expression of humanized Immuno-STAT-IL2.FH_4. Reducing (R) and non-reducing (NR) SDS-PAGE of the humanized Immuno-STAT-IL2.FH_4 framework bearing either two copies of CMV $pp65_{495-503}$/HLA-A*0201 or two copies of MelanA/$MART1_{26-35}$/HLA-A*0201 genetically fused to four copies of IL-2(F42A, H16A).

Supplementary Figure 8

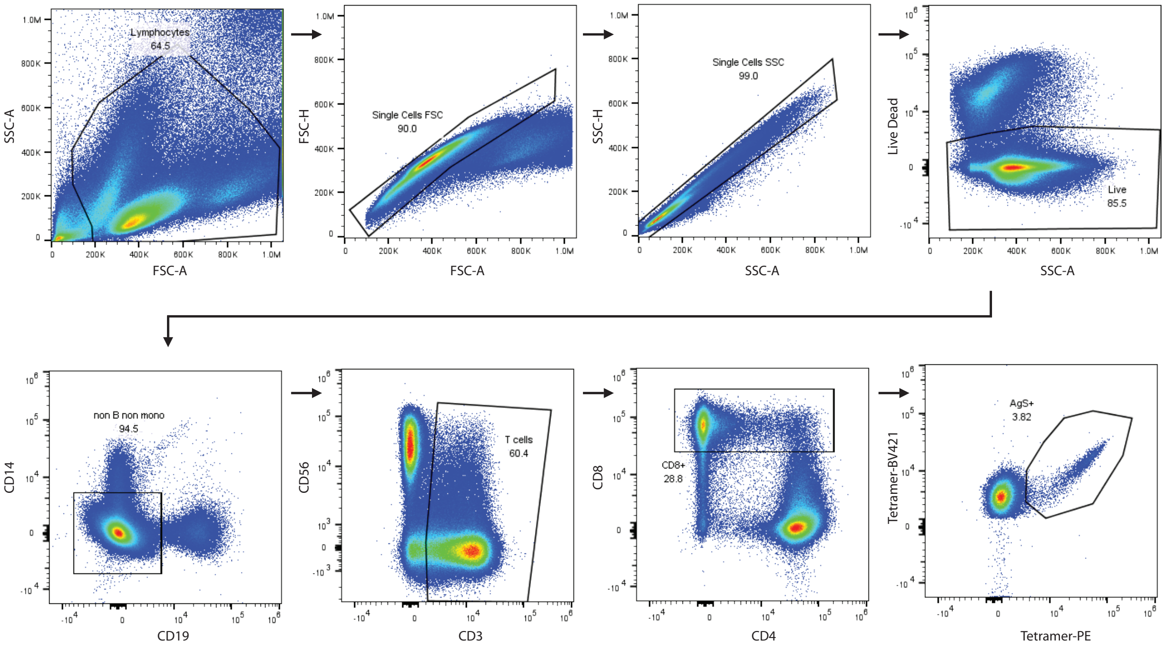

Supplementary Figure 8. Gating strategy for measuring IST-driven in vitro expansions of antigen-specific T cells from donor PBMC. Representative gating strategy and FACS analysis of 10 day in vitro expanded CMV pp65$_{495-503}$/HLA-A*0201-specific T cells from antigen-reactive donor PBMC in the presence of CMV-IST-IL2.FH$_4$. CMV pp65$_{495-503}$/HLA-A*0201-specific CD3$^+$CD8$^+$CD14$^-$CD19$^-$ T cells were enumerated by co-staining with APC-labeled CMV pp65$_{495-503}$/HLA-A*0201 tetramers and with PE-labeled tetramers of the same specificity.

Supplementary Figure 9

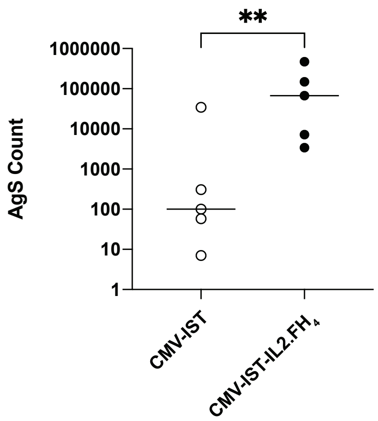

Supplementary Figure 9. Dependence of Immuno-STAT framework on IL2.FH_4 for proliferation of human AgS T cells. CMV-specific human PBMC T cell counts following ten day culture with 10 nM CMV-IST-IL2.FH_4 or 10 nM CMV-IST (pHLA-Fc without IL2.FH_4). Data represent single measurements per condition from 5 donors. Statistical significance ** ($P = 0.0028$) assessed by one-tailed paired ratio T test.

Supplementary Figure 10

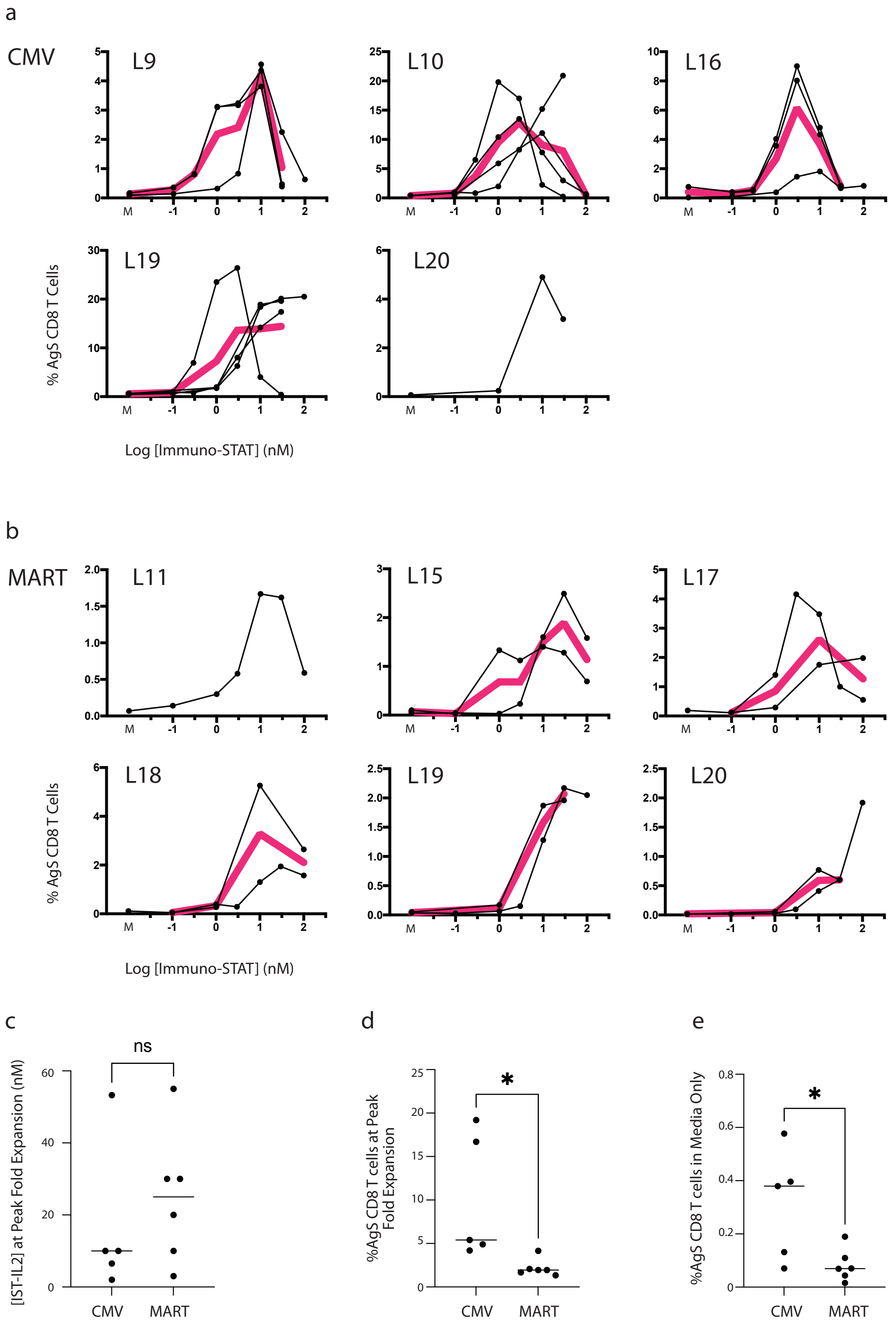

Supplementary Figure 10. CMV-IST-IL2.FH_4 and MART-IST-IL2.FH_4 expansion detail. (a) In vitro proliferation for PBMC from donors L9, L10, L16, L19, and L20 in response to increasing concentrations of CMV-IST-IL2.FH_4. Thin solid lines represent individual expansion trials per donor. Thick red line represents average for repeated doses in donors with multiple expansion trials; M = media alone. (b) As in (a) for donors L11, L15, L17, L18, L19, and L20 in response to increasing concentrations of IST-MART-IL2.FH_4 or media alone. (c) IST-IL2.FH_4 concentration providing the greatest fold expansion in CMV-IST-IL2.FH_4 and IST-MART-IL2.FH_4 expansions. Bar represents median value. Difference between CMV and MART groups is not significant (ns) by two-tailed unpaired t-test (P = 0.5002). (d) Percent antigen-specific (AgS) CD8 T cells at peak fold expansion. Bar represents median value. P = 0.0259 (*) by two-tailed unpaired t-test. (e) Percent AgS CD8 T cells measured for cells incubated in media only. Bar represents median value. P = 0.0297 (*) by two-tailed unpaired t-test.

Supplementary Figure 11

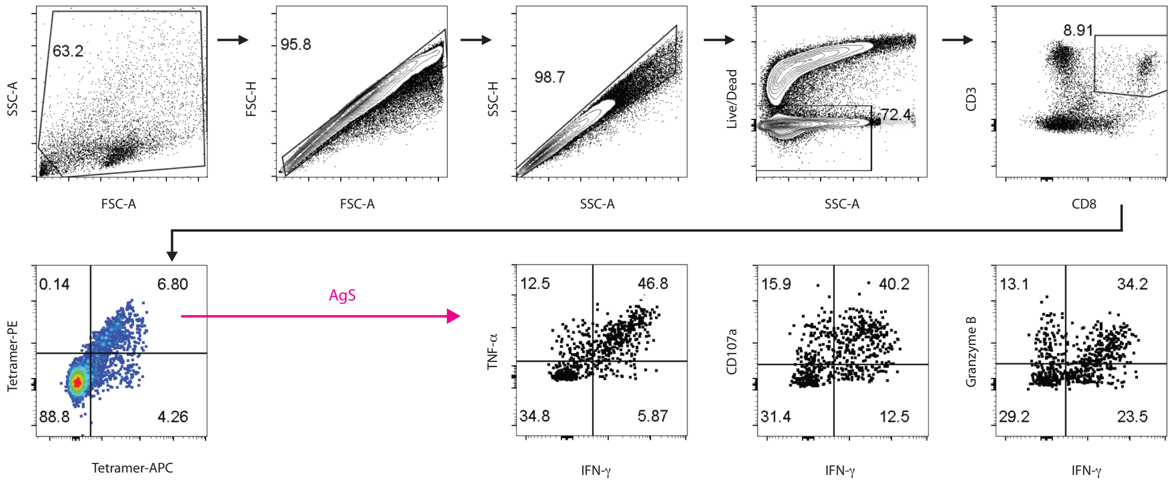

Supplementary Figure 11. Gating strategy for assessing IFN-γ, TNF-α, CD107a, and granzyme B expression in peptide-expanded and Immuno-STAT-IL2.FH$_4$-expanded AgS CD8 T cells. Representative gating strategy and FACS analysis of peptide-expanded MART1$_{26\text{-}35}$/HLA-A*0201-specific T cells assessed for IFN-γ, TNF-α, CD107a, and granzyme B expression.

Supplementary Figure 12

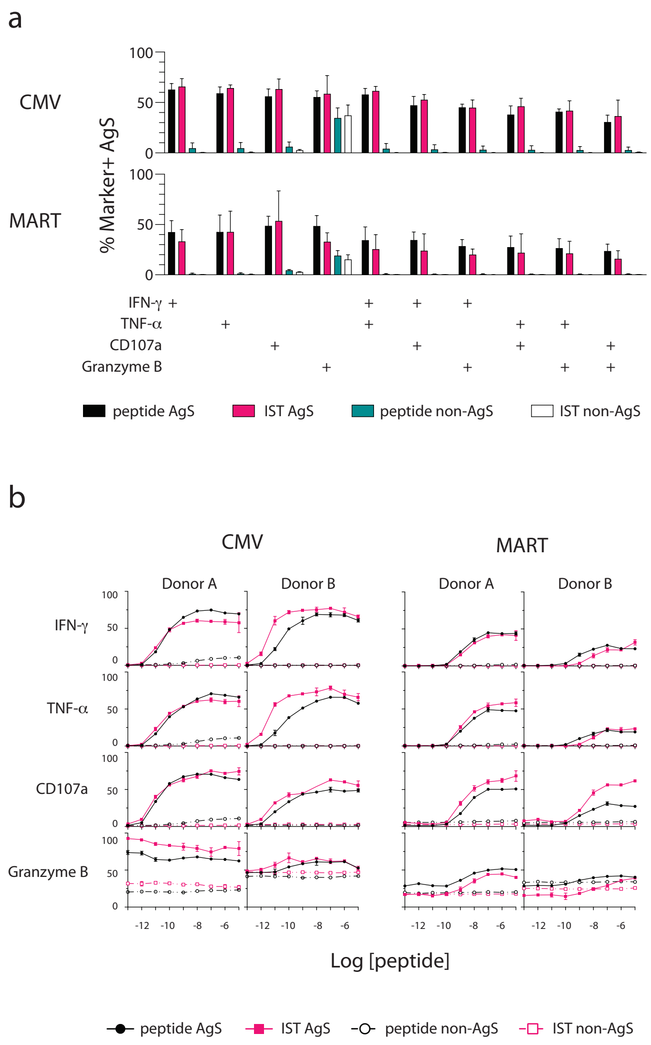

Supplementary Figure 12. Quantitation of IFN-γ, TNF-α, CD107a, and granzyme B for peptide-expanded and Immuno-STAT-IL2.FH_4-expanded AgS CD8 T cells challenged with cognate or irrelevant peptide. (a) Frequencies of cognate peptide-expanded or specific Immuno-STAT-IL2.FH_4-expanded CD8 T cells from human PBMC which are single or double positive for IFN-γ, TNF-α, CD107a, or granzyme B following challenge with cognate peptide or irrelevant peptide HIV-1 p17 Gag $_{77-85}$ (SLYNTVATL; SL9). Peptide AgS or peptide non-AgS refers to peptide-

expanded PBMC challenged with cognate or irrelevant peptide, respectively. IST AgS or IST non-AgS refers to specific-Immuno-STAT-IL2.FH_4-expanded PBMC challenged with cognate or irrelevant peptide, respectively. Data represent mean $\pm$ s.d. from duplicate samples of three different donors. AgS CD8 T cells are defined as the double tetramer positive population described in Supplementary Figure 11. (b) Frequencies of marker positive cells among CMV-IST-IL2.FH_4 or MART-IST-IL2.FH_4 expanded AgS CD8 T cells as a function of challenge peptide concentration. Data are mean $\pm$ s.d. of triplicate samples from each donor shown.

Supplementary Figure 13

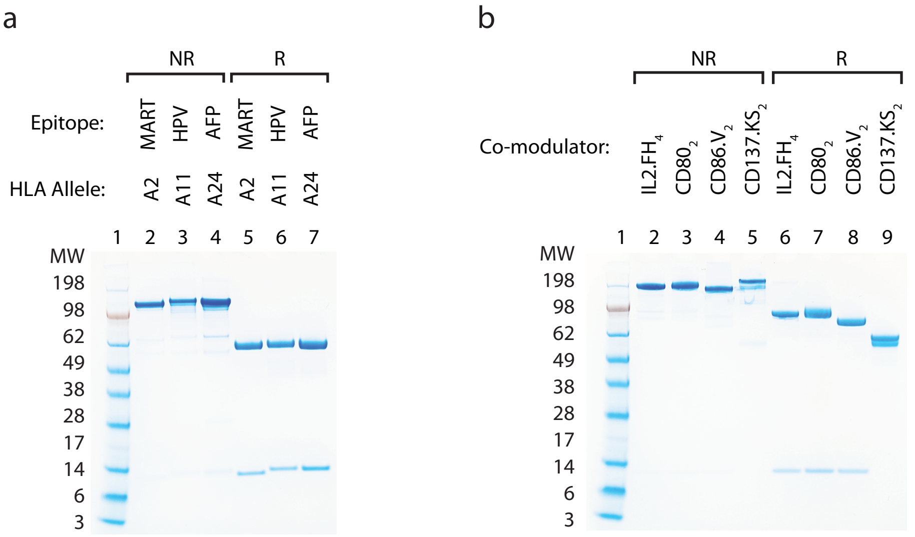

Supplementary Figure 13. Alternative HLA alleles and co-modulators on the Immuno-STAT framework. (a) Reducing (R) and non-reducing (NR) SDS-PAGE of $AFP_{403-411}$/HLA-A*1101 and HBV ayw $P_{109-118}$/HLA-A*2402 on the Immuno-STAT framework, pHLA-Fc only. $MART_{26-35}$/HLA-A*0201 (Fc only) included as a reference. (b) Reducing (R) and non-reducing (NR) SDS-PAGE of MART-IST bearing either four copies of IL2.FH ($IL2.FH_4$) or two copies of the CD80 ectodomain ($CD80_2$; fused to N-terminus of HLA-Fc) or two copies of the CD86 IgV domain ($CD86.V_2$; fused to N-terminus of HLA-Fc) or two copies of single-chain homotrimeric CD137L, K127A mutant ($CD137L.KS_2$; fused to C-terminus of β2m).

Supplementary Figure 14

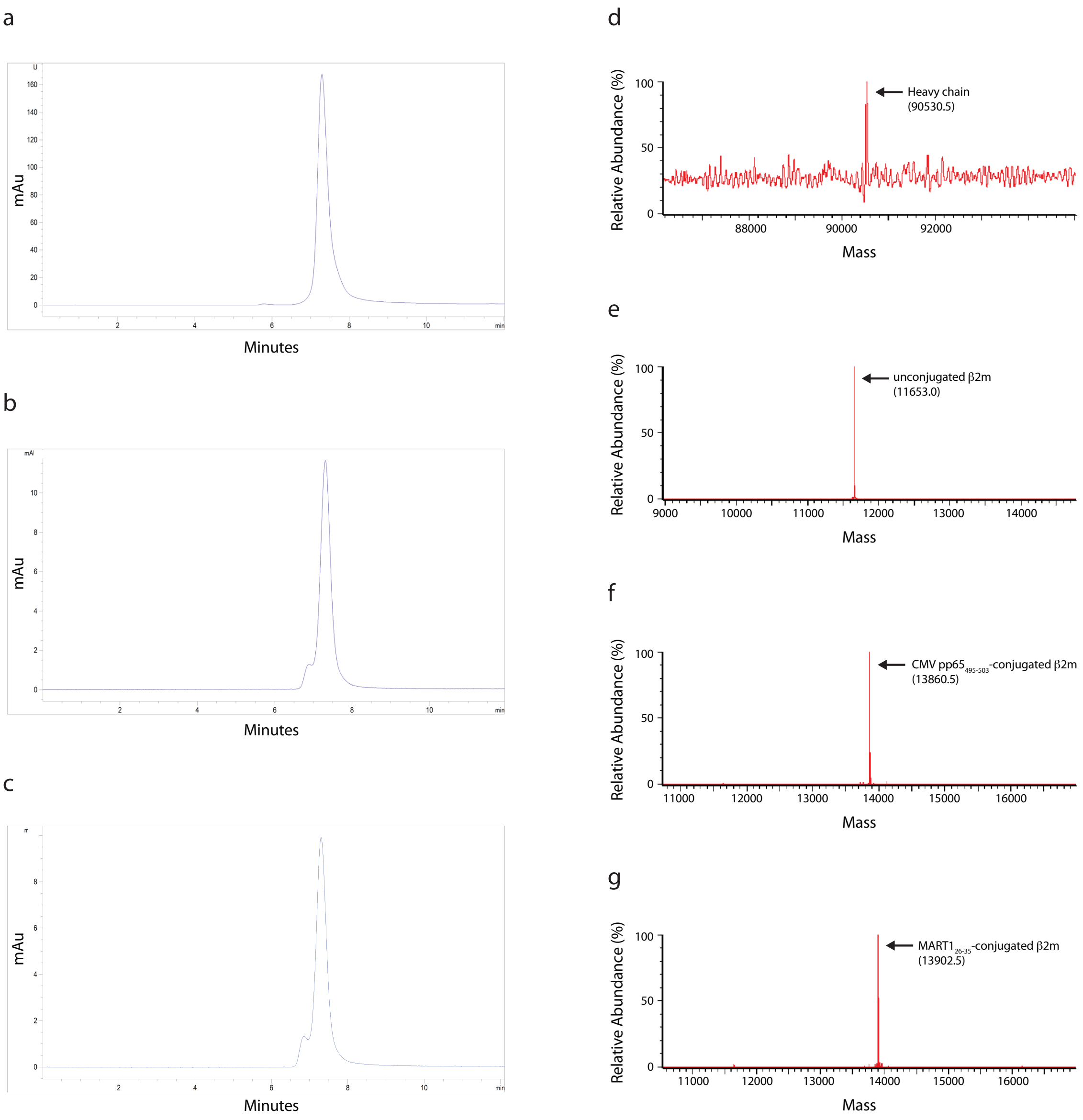

Supplementary Figure 14. Neo-STAT conjugation and purification. Analytical size exclusion chromatography demonstrating stable monomeric solution state of (a) unconjugated, (b) CMV pp65$_{495\text{-}503}$ peptide-conjugated, or (c) MART1$_{26\text{-}35}$ peptide-conjugated Neo-STAT molecules. Reduced intact mass analysis using electrospray ionization time of flight mass spectrometry

(ESI-TOF MS) confirming intact (d) Neo-STAT HLA-A*0201 heavy chain and (e) unconjugated Neo-STAT β2m. ESI-TOF MS analysis demonstrating Neo-STAT β2m mass shift corresponding to (f) CMV pp65$_{495\text{-}503}$ peptide-conjugated Neo-STAT β2m and (g) MART1$_{26\text{-}35}$ peptide-conjugated Neo-STAT β2m.

Supplementary Tables

Supplementary Table 1. LCMV-Immuno-STAT IL-2 Variants Screen and Ranking

	IL-2 Mutations											Ranking			
IL-2 Copy No.	F42A	H16A	D20K	Y45A	E15A	Q126	$logEC50_{P14}$ (nM)	$logEC50_{B6}$ (nM)	$logEC50_{B6}$ - $logEC50_{P14}$	Δ Signal at $EC50_{P14}$ (%)	Expression Titer (mg/L)	Rank logEC50	Rank Δ Signal at $EC50_{P14}$	Rank Titer	Average Rank
4	+	+					0.968	2.551	1.583	20.7	105	7	5	2	4.7
4	+						0.414	2.225	1.811	22.3	69	3	3	10	5.3
2	+						1.02	2.52	1.503	22.2	89	9	4	5	6.0
4							0.740	2.434	1.695	24.1	61	5	1	14	6.7
2	+	+					1.89	3.05	1.163	18.8	107	14	6	1	7.0
2							1.38	2.81	1.429	23.1	73	12	2	8	7.3
2	+		+				1.01	2.53	1.522	3.70	71	8	10	9	9.0
2	+	+				+	0.762	1.436	0.673	4.30	62	6	9	13	9.3
4	+	+				+	2.08	2.46	0.383	4.42	82	16	8	6	10.0
2	+		+			+	0.364	1.968	1.604	2.93	36	2	11	19	10.7
2	+	+	+				2.61	3.04	0.433	9.07	78	21	7	7	11.7
4	+		+				0.359	2.475	2.116	1.51	31	1	17	20	12.7
4	+	+	+	+			1.98	2.93	0.945	1.30	93	15	19	4	12.7
2	+		+		+		1.03	3.01	1.981	2.45	53	10	12	16	12.7
2	+		+	+			2.14	3.53	1.387	1.79	67	18	14	11	14.3
4	+		+			+	0.556	1.913	1.357	1.36	17	4	18	22	14.7
2	+	+	+	+		+	2.13	3.87	1.742	1.54	66	17	16	12	15.0
4	+	+	+				3.08	3.18	0.101	0.73	99	22	20	3	15.0
2	+		+	+		+	2.29	2.56	0.267	1.76	61	20	15	14	16.3
2	+	+	+	+			2.24	3.38	1.140	2.22	39	19	13	18	16.7
4	+	+	+	+		+	1.39	2.72	1.330	0.0731	50	13	22	17	17.3
4	+		+	+		+	1.23	2.93	1.705	0.07	19	11	21	21	17.7

Supplementary Table 1. Immuno-STAT-IL-2 Variants Screen and Ranking.

$logEC50_{P14}$, Δ signal at $EC50_{P14}$, and protein expression titer values and rankings for IL-2 variants on the LCMV-Immuno-STAT framework corresponding to heat map shown in Supplementary Figure 3. Pre-normalized values, component ranks and average ranks for each Immuno-STAT are shown.

Supplementary Table 2. Δ LogEC50 and Δ pSTAT5 Signal for Top-Ranked Immuno-STAT-IL2 Frameworks

Construct	No. IL-2/ Molecule	IL-2 Variant	$LogEC50_{P14}$	$LogEC50_{B6}$	Δ LogEC50	Δ Signal at $EC50_{P14}$ (%)
LCMV-IST-IL2.FH_4	4	F42A H16A	0.563 ± 0.366	2.22 ± 0.250	1.66 ± 0.119	49.2 ± 0.213
LCMV-IST-IL2.F_4	4	F42A	-0.326 ± 0.278	1.40 ± 0.134	1.72 ± 0.156	48.7 ± 0.594
LCMV-IST-$IL2_4$	4	WT	-0.593 ± 0.662	1.42 ± 0.510	2.02 ± 0.174	48.9 ± 0.489
LCMV-IST-$IL2_2$	2	WT	0.272 ± 0.667	1.83 ± 0.325	1.56 ± 0.349	47.3 ± 1.72
IL2.FH_4-Fc	4	F42A H16A	1.77 ± 0.385	1.81 ± 0.378	0.0373 ± 0.0152	2.49 ± 1.15
rhIL-2	1	WT	1.80 ± 0.266	1.94 ± 0.109	0.144 ± 0.179	7.43 ± 8.40

Supplementary Table 2. Δ logEC50 and Δ pSTAT5 signal for top-ranked Immuno-STAT-IL2 variants. $LogEC50_{P14}$, $logEC50_{B6}$, Δ logEC50 and Δ pSTAT5 signal at $EC50_{P14}$ for top-ranked Immuno-STAT-IL2 variants and controls from Figure 1b and Supplementary Figure 4. Data represent means ± s.d. of duplicate samples from three independent experiments.

Supplementary Table 3. Predicted Peptide Affinity to HLA-A*0201 for Expressible and Non-Expressible Epitopes on IST-IL2.FH_4 Framework

Protein	Subsequence (Variant)	Epitope Sequence	Peptide Length	Predicted Affinity (nM)	Expressed
HPV E7	11-20	YMLDLQPETT	10	44	Yes
AFP	158-166	FMNKFIYEI	9	2	Yes
BALF4	276-284	FLDKGTYTL	9	4	Yes
CEA	571-579	YLSGANLNL	9	13	Yes
CMV pp65	495-503	NLVPMVATV	9	29	Yes
FLU-M1	58-66	GILGFVFTL	9	12	Yes
gp100	209-217	IMDQVPFSV	9	6	Yes
gp100	154-162	KTWGQYWQV	9	9	Yes
HBV Core	18-27	FLPSDFFPSV	10	4	Yes
Her2/neu	369-377 (I370V, L377V)	KVFGSLAFV	9	10	Yes
LMP1	125-133	YLLEMLWRL	9	2	Yes
MAGE-A10	254-262	GLYDGMEHL	9	9	Yes
MAGE-A3	112-120	KVAELVHFL	9	12	Yes
MAGE-A4	230-239	GVYDGREHTV	10	393	Yes
MelanA/Mart	26-35	ELAGIGILTV	10	137	Yes
NY-ESO-1	157-165, C165A	SLLMWITQA	9	23	Yes
NY-ESO-1	157-165, C165V	SLLMWITQV	9	6	Yes
p53	264-272	LLGRNSFEV	9	17	Yes
PAP-3	135-143	ILLWQPIPV	9	6	Yes
PAP-3	11-19	FLGYLILGV	9	6	Yes
PSA1	141-150	FLTPKKLQCV	10	37	Yes
PSMA	4-12	LLHETDSAV	9	56	Yes
PSMA	27-38	VLAGGFFLL	9	6	Yes
PSMA	711-719	ALFDIESKV	9	13	Yes
PSMA	663-671	MMNDQLMFL	9	4	Yes
Survivin	96-104	LMLGEFLKL	9	34	Yes
Tyrosinase	369-377, 371D	YMDGTMSQV	9	6	Yes
WT1	126-134	RMFPNAPYL	9	7	Yes
AFP	137-145	PLFQVPEPV	9	240	No
BCR-ABL	n/a	GVRGRVEEI	9	17441	No
CEA	694-702	GVLVGVALI	9	833	No
EBV BMLF-1	280-288	GLCTLVAML	9	112	No
EGFR	1-9 (variant 3)	LEEKKGNYV	9	26589	No
KRAS	5-14 (G12D)	KLVVGADGV	9	262	No
LMP2	356-364	FLYALALLL	9	10	No
LMP2	426-434	CLGGLLTMV	9	69	No
MUC1	950-958	STAPPVHNV	9	487	No
NY-ESO-1	157-165	SLLMWITQC	9	1015	No
p53	149-157	STPPPGTRV	9	13197	No

Supplementary Table 3. Predicted peptide affinity to HLA-A*0201 for expressible and non-expressible epitopes on the Immuno-STAT-IL2.FH_4 framework. Affinities as predicted by NetMHC3.4 (http://www.cbs.dtu.dk/services/NetMHC-3.4/).
